# Acalabrutinib treatment in relapsed/refractory primary cutaneous diffuse large B cell lymphoma, leg type. Case report

**DOI:** 10.3389/fonc.2026.1771103

**Published:** 2026-03-16

**Authors:** David A. Martinez-Gamboa, Flavio F. Beas, Eider Moreno-Cortes, Leah A. Swanson, Aaron R. Mangold, Fabio Vargas, Januario E. Castro

**Affiliations:** 1 Division of Hematology and Medical Oncology, Mayo Clinic, Phoenix, AZ, United States; 2 Cancer Research and Cellular Therapies Laboratory, Mayo Clinic, Phoenix, AZ, United States; 3 Department of Internal Medicine, Mayo Clinic, Phoenix, AZ, United States; 4 Department of Dermatology, Mayo Clinic, Phoenix, AZ, United States; 5 Department of Laboratory Medicine and Pathology, Mayo Clinic, Scottsdale, AZ, United States

**Keywords:** acalabrutinib, Bruton’s tyrosine kinase, case report, ibrutinib, primary cutaneous diffuse large B-cell lymphoma-leg type

## Abstract

**Background:**

Primary cutaneous diffuse large B-cell lymphoma, leg type (PCDLBCL-LT), is a rare subset of primary cutaneous lymphoma known for its aggressive behavior, poor prognosis, and limited treatment options, particularly in the relapsed or refractory setting. We report a case of a patient diagnosed with PCDLBCL-LT who achieved a clinically significant response and sustained remission for seven months with acalabrutinib, a covalent inhibitor of Bruton’s tyrosine kinase (BTK), before disease progression.

**Case presentation:**

An 88-year-old woman presented with a rapidly enlarging ulcerated lesion on the left lower leg. Histopathological and immunohistochemical analyses confirmed the diagnosis of PCDLBCL-LT. She was treated with rituximab, cyclophosphamide, doxorubicin, vincristine, and prednisone (R-CHOP) chemoimmunotherapy, followed by external beam radiation therapy (RT). These interventions resulted in only a partial response, and the disease progressed within several months. The patient was receiving direct oral anticoagulant (DOAC) therapy for left lower limb deep vein thrombosis, and she also had a history of hypertension. Considering the increased risk of major bleeding associated with DOAC use and the elevated risk of cardiovascular adverse events previously reported with ibrutinib, an alternative BTK inhibitor was considered. Accordingly, she was initiated on acalabrutinib at a dose of 100 mg orally twice daily. The patient exhibited a rapid and robust clinical response, with complete resolution of cutaneous lesions and sustained remission lasting seven months. Ultimately, the disease progressed, and given her advanced age and overall poor performance status, she was transitioned to hospice care. She subsequently died 14 months after initiating acalabrutinib.

**Conclusion:**

This report documents the first known case of successful treatment of relapsed/refractory PCDLBCL-LT with acalabrutinib. Despite advanced age and poor performance status, the patient experienced rapid symptom resolution and durable remission without treatment-related adverse events. These findings suggest that BTK inhibitors may represent a promising therapeutic approach for PCDLBCL-LT, either as monotherapy or in combination with anti-CD20 antibodies. Further investigation into novel targeted therapies is urgently needed for this rare and aggressive lymphoma subtype.

## 1 Introduction

Primary cutaneous diffuse large B-cell lymphoma, leg type (PCDLBCL−LT), is a rare and clinically aggressive subtype of primary cutaneous B-cell lymphoma (PCBCL), accounting for approximately 10-20% of PCBCLs (1). The disease predominantly affects older adults, with a median age at diagnosis of approximately 70 years, and shows a female predominance (female-to-male ratio ~2:1) (2). Recognized as a distinct entity in both the 2018 WHO classification and the International Consensus Classification (3–5), PCDLBCL−LT typically manifests as rapidly enlarging erythematous to violaceous nodules or plaques on the lower extremities, although 10–20% of cases involve other anatomical sites (2).

Histopathologically, PCDLBCL−LT is characterized by diffuse dermal infiltration of large atypical centroblasts and immunoblasts, typically with high mitotic activity and a paucity of reactive T cells (6, 7). Immunophenotypically, tumor cells consistently express pan–B-cell markers, including CD20, CD19, CD22, CD79a, and PAX5, and are characteristically positive for BCL2, MUM1/IRF4, and FOXP1, while generally negative for germinal center markers such as CD10 and CD5 (6, 7). At the molecular level, PCDLBCL−LT most often exhibits an activated B-cell–like (ABC) gene expression profile. Recurrent oncogenic mutations in MYD88 and CD79B are common, particularly the MYD88 L265P variant, which promotes constitutive NF−κB activation and contributes to the disease’s aggressive clinical behavior (2, 8).

Standard treatment for PCDLBCL-LT involves rituximab-based chemoimmunotherapy, such as R-CHOP (rituximab, cyclophosphamide, doxorubicin, vincristine, and prednisone), often supplemented with involved-site radiotherapy (ISRT). Although initial complete remission (CR) rates are high, relapse is common, occurring in approximately 70% of cases, and the 5-year disease-specific survival remains around 50% (9). Management of relapsed or refractory disease is particularly challenging in elderly or comorbid patients who may not be candidates for intensive cytotoxic regimens. Recent advances in targeted therapies have expanded treatment options for B-cell malignancies. Among these, Bruton’s tyrosine kinase inhibitors (BTKis) have shown significant efficacy in non-Hodgkin lymphomas (NHLs), particularly in those with ABC phenotypes (10, 11). Ibrutinib, a first-generation covalent BTKi, has demonstrated anecdotal efficacy in PCDLBCL-LT, though published data remain limited. The role of second-generation BTKis, such as acalabrutinib, has not been well characterized in this specific subtype.

Here, we report the case of an 88-year-old woman with relapsed/refractory PCDLBCL-LT who achieved a rapid clinical response and sustained remission for seven months following treatment with acalabrutinib, a selective covalent BTK inhibitor. This case highlights the potential role of BTK-targeted therapies in the management of PCDLBCL-LT. It underscores the need for further investigation of novel, tolerable treatment options for this aggressive cutaneous lymphoma.

## 2 Case presentation

An 88-year-old woman with a remote history of stable chronic lymphocytic leukemia (CLL), diagnosed 23 years prior, presented to the outpatient oncology clinic with a small erythematous lesion on the posterior left. Core needle biopsy demonstrated CD5+ diffuse large B-cell lymphoma (DLBCL), raising concern for Richter’s transformation. However, staging with positron emission tomography (PET) revealed isolated hypermetabolic activity confined to the left lower leg without systemic involvement, findings consistent with a primary cutaneous origin.

The patient initially received rituximab with prednisone, which produced no clinical response. She was subsequently treated with six cycles of R-CHOP chemoimmunotherapy. Four months after initiation, restaging PET-CT demonstrated only a partial metabolic response, with persistent subcutaneous nodules in the left leg. To address residual disease, she underwent external beam radiation therapy (RT), receiving 4000 cGy over several weeks, which achieved a partial response (PR). Shortly after completing RT, she developed a left lower extremity deep venous thrombosis (DVT) and was started on apixaban.

**Figure 1:**
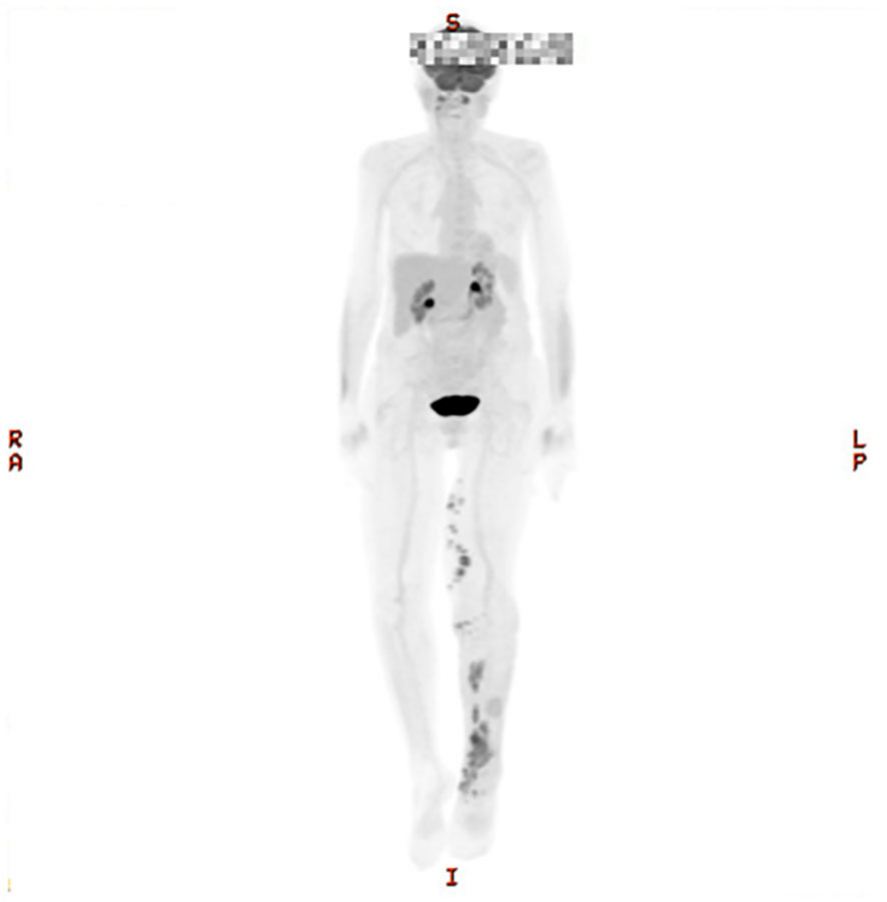
PET-scan imaging showing confluent and clustered hypermetabolic lesions localized to the medial aspect of the left lower extremity. With cutaneous uptake extending from the proximal medial thigh and calf down to the medial aspect of the left foot.

**Figure 2:**
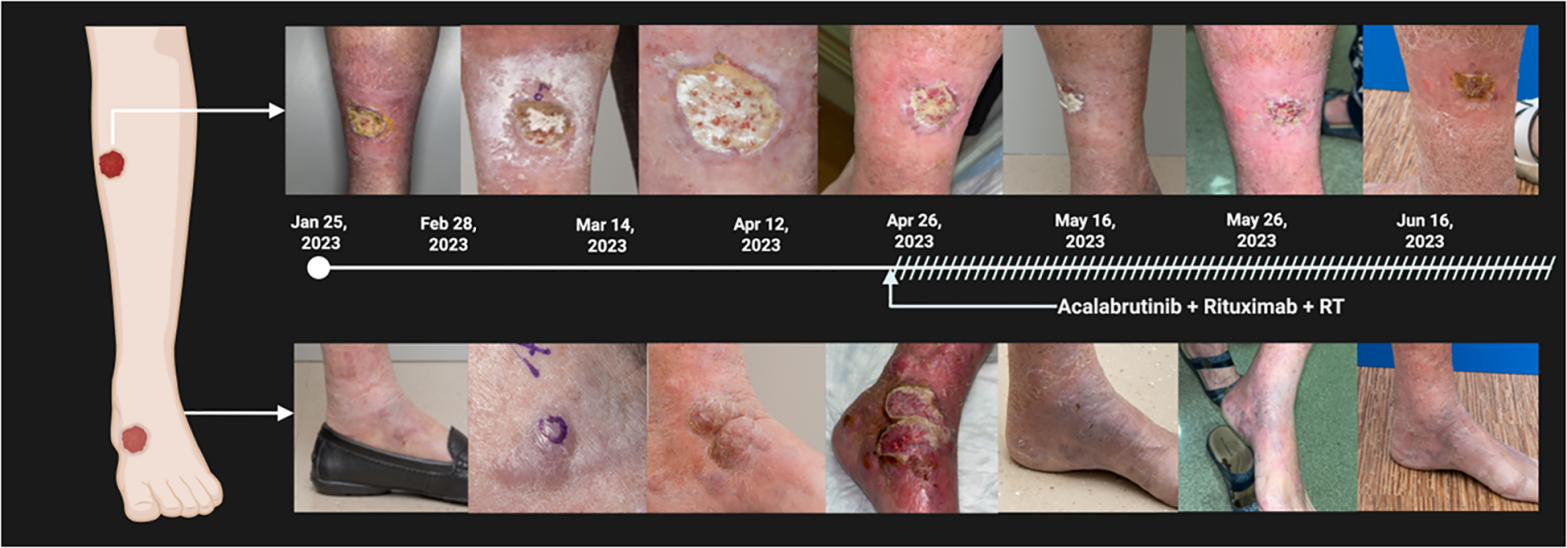
Clinical progression and response of PCDLBCL-LT to combination therapy with acalabrutinib, Rituximab, and localized radiotherapy, Timeline of cutaneous lesions on the left lower extremity from initial presentation (January 25, 2023) to follow-up June 16, 2023. Serial photographs show marked clinical improvement, with significant reduction in lesion size, re-epithelization, and near complete resolution by June 2023. The upper row correspond to the anterior leg lesion; the lower row shows the lateral ankle lesions.

Nine months after initial diagnosis, new violaceous nodules appeared on the medial foot and upper calf. New imaging confirmed disease progression with multiple new hypermetabolic cutaneous lesions, consistent with relapse. At 11 months post-diagnosis, a follow-up PET scan revealed further progression with new confluent hypermetabolic clusters and persistent inguinal lymphadenopathy (**Figure 1**). A repeat biopsy was performed, showing a dense dermal infiltrate of large atypical lymphoid cells with round nuclei. Immunohistochemistry (IHC) demonstrated positivity for CD20, PAX5, BCL2, MUM1, CD5, LEF1, and MYC, with negative staining for CD10, BCL6, CD3, CD23, Cyclin D1, EBV ISH, and TdT (**Figure 2**). Fluorescence *in situ* hybridization (FISH) identified a MYC/IGH rearrangement. These findings confirmed the diagnosis of CD5+ PCDLBCL-LT.

Given the patient’s rapidly progressive and symptomatic disease, systemic therapy was reinitiated. Following multidisciplinary review, rituximab in combination with acalabrutinib was selected, followed by palliative local RT. Acalabrutinib, a second-generation Bruton’s tyrosine kinase inhibitor (BTKi), was chosen due to its improved safety profile relative to ibrutinib, particularly a lower reported incidence of cardiovascular toxicity and major bleeding events which is an important consideration given the patient’s concurrent DOAC therapy (12, 13).

**Figure 3:**
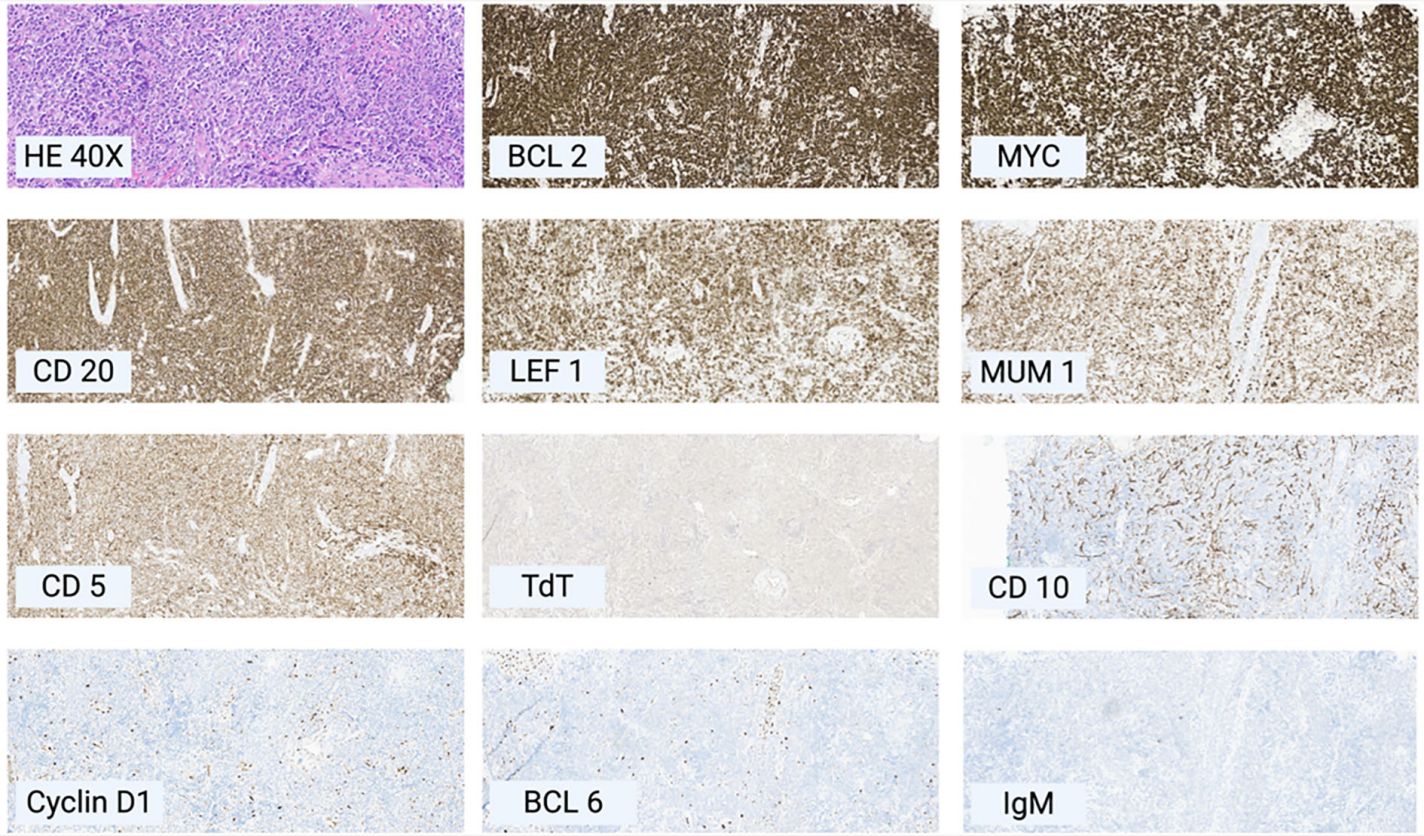
Immunohistochemical profile of patient’s cutaneous biopsy on left leg lesion showing features consistent with PCDLBCL-LT, magnification 20X unless otherwise specified. All stains performed on formalin-fixed-paraffin-embedded sections. BCL2+, MYC+, CD20+, LEF1+, MUM1+, CD5+, TdT-, CD10-, Cyclin D1-, BCL6.

After one month of acalabrutinib therapy, the patient experienced marked clinical improvement, including decreased edema, flattening of nodular lesions, and resolution of pain (**Figure 3**). Restaging PET-CT demonstrated a complete metabolic response (CR). She maintained remission for seven months without evidence of recurrence. Ultimately, disease relapsed with new cutaneous lesions and progressive functional decline. Given her age, comorbidities, and expressed wishes, no further systemic treatment was pursued. She was transitioned to hospice care with an emphasis on comfort and quality of life.

## 3 Discussion

This case illustrates the diagnostic and therapeutic challenges associated with PCDLBCL-LT, a rare and aggressive subtype of PCBCL. The disease typically presents as rapidly enlarging tumors on the lower extremities, as in our patient, with localized cutaneous involvement restricted to the lower leg. The clinical course is usually aggressive without systemic dissemination (which is observed in approximately one-third of cases during the disease course) (9, 14).

The patient showed characteristic histological structures of PCDLBCL-LT with diffuse sheets of large, transformed B cells with centroblastic or immunoblastic morphology. Immunophenotypically, most PCDLBCL-LT cells express pan-B-cell markers (CD20, CD79a, PAX5), along with MUM1, BCL2, IgM, and FOX1. BCL6 is usually dimly expressed, while CD3, CD10, and CD5 are typically negative (6).

Our case was unusual in demonstrating aberrant CD5 positivity, a rare finding reported in only a small number of cases (n=8 to date (15–22)). CD5 and LEF1 expression in this patient might reflect an underlying relationship with the patient’s known chronic lymphocytic leukemia (CLL). Large-cell Richter transformation was considered in the differential diagnosis, but the clinical and ancillary study findings supported a primary cutaneous large B-cell lymphoma. The prognostic implications and treatment response of CD5 expression remain unclear. The selection of a BTKi in this case, was made in part, as an attempt to treat the possibility of CLL/Richter’s involvement.

Clonality studies could not be performed on the original diagnostic material. Interestingly, FISH analysis in our case demonstrated a MYC/IGH fusion with absence of BCL2 or BCL6 rearrangements. Most PCDLBCL-LT tumors are characterized by a high proliferative index (Ki-67 >50%) and a non-germinal center B-cell phenotype (6). At the molecular level, MYC expression is observed in 65–80% of cases (21), and recurrent mutations in MYD88 and CD79B (4, 23). Our patient’s tumor also demonstrated evolving immunophenotypic changes, including decreased expression of CD20, CD43, and CD5 after therapy, consistent with treatment-induced modulation following five months of targeted therapy.

This patient received the standard first-line treatment for PCDLBCL-LT which included rituximab-based chemotherapy, R-CHOP. This regimen has significantly improved disease-specific survival from 30-50% to 70-80% and overall survival compared to non-rituximab-based therapies (9, 14). In patients with localized disease, or those unfit for systemic therapy, involved-site radiation therapy (ISRT) is recommended. Importantly, the combination of ISRT with R-CHOP is associated with superior outcomes, with reported improvements in both progression-free survival (41.8 *vs*. 13.7 months) and overall survival (74.8 *vs*. 38.2 months) (24).

Despite the use of intense chemoimmunotherapy and radiation therapy, these tumors often disseminate and relapse with a high risk of generalized skin lesions causing open wounds, as we observed in our case (24, 25). Relapses in PCDLBCL-LT are common and challenging, often manifesting as generalized cutaneous disease (24). There are no FDA-approved therapies specifically for relapsed PCDLBCL-LT, and management is extrapolated from systemic DLBCL treatments (26, 27). Investigational and off-label approaches include, and small molecule inhibitors such as lenalidomide, Venetoclax, and BTKi (26–28).

BTK plays a central role in B-cell receptor (BCR) signaling, driving proliferation and survival in malignant B cells. BTKi disrupt this signaling and reduce tumor–microenvironment interactions, ultimately promoting cell death (29). BTKi are particularly effective in B-cell malignancies harboring MYD88 mutations, such as CLL, mantle cell lymphoma, and marginal zone lymphoma (29). There are no randomized or controlled studies reporting the use of BTKi in PCDLBCL-LT; however, in the literature, we found eight case reports documenting the use of BTKi in the treatment of relapsed or refractory PCDLBCL-LT (30–35). (**Table 1**) Notably, Gupta et al. reported a case in which a patient achieved a sustained CR after two years of follow-up through a combination of RT, lenalidomide, and ibrutinib (32). Likewise, Moore et al. presented two cases where a regimen involving lenalidomide, ibrutinib, and rituximab was chosen based on the presence of the MYD88 (L265P) mutation. Both patients achieved CR after four cycles and maintained this response for nine and twenty months (30). The specific mutation of MYD88 (L265P) is associated with a favorable response to BTKi and has been documented in a case report by Pang et al. (33).

**Table 1:** Reported cases of PCDLBCL-LT Treated with BTK inhibitor-Based Therapies, summarizing demographics, prior treatments, histopathological and molecular features, therapeutic regimens, clinical response and duration of response.

Author	Year	Age, gender	Prior lines of therapy	Histological phenotype	Treatment	Outcome achieved	Duration of response
Moor et al. (30)	2022	78 y/o M	R-CHOP, mini-R-CHOP, RT	CD20+, BCL2+, MUM1+, CD10-, CD5-, CD30-, BCL6-, Ki-67 60% FISH (Negative for MYC, BCL2, BCL6 rearrangements). * MYD88 L265P mutation	Ibrutinib 560 mg + Lenalidomide 25 mg + Rituximab 375 mg/m2 (6 cycles) Ibrutinib as maintenance	CR	9 mo †
		61 y/o M	R-CHOP	CD20+, BCL2+, FOXP1+ , IgM +, MYC+, MUM1 +/-, CD30-, CD10-, Cyclin D1-, PD1-, PD-L1-. FISH (Negative for BCL2, BCL6 rearrangements) *MYD88 L265P mutation	Ibrutinib 560 mg + Lenalidomide 20 mg + Rituximab 375 mg/m2 (6 cycles) Ibrutinib + Lenalidomaide maintenance	CR	20 mo †
Fox et al. (31)	2018	80 y/o F	R-CHOP, Low doseR- GemVin + RT	CD20+, PAX5+, FOXP1+, CD5+, BCL2+, MUM1+, CD10-, BCL6- * MDY88 L265P mutation, CD79B Y196H mutation	Ibrutinib 420 mg	CR	5 mo →
Gupta et al. (32)	2015	62 y/o M	R-CHOP + RT, R-ICE, Bexxar-BEAM, SCT, Lenalidomide	CD20+, CD79a+, BCL2+, MUM1+, CD10-, BCL6-	Ibrutinib 560 mg	CR	24 mo →
Pang et al. (33)	2019	56 y/o M	R-CHOP + RT, Obinutuxzumab, Lenalidomide	CD20+, BCL2+, BCL6+, FOXP1+	Ibrutinib 140 mg	CR	32 mo →
Al-Obaidi et al. (34)	2020	84 y/o M	R-CVP + RT, R-Bendamustine, R-Lenalidomide	CD20+, CD43+, PAX5+, MUM1+, BCL2+, BCL6+/-, CD3-, CD5-, CD10-, CD21-, CD23-, CD30-, BCL1-, EBER-. Ki-67 75%. FISH (Negative for MYC)	Ibrutinib	CR	8 mo →
Deng et al. (35)	2017	70 y/o F	R-CHP, R-EPCH, R-ICE, Lenalidomide	CD20+, CD79a+, BCL2+, BCL6+, MUM1+, CD10-. *MYD88 L265P mutation CD79 WT	Ibrutinib 560 mg + R-EPOCH (4 cycles) Ibrutinib as maintenance	CR	20 mo →

CR, Complete response; RT, Radiotherapy; R-CHOP, Rituximab, Cyclophosphamide, Doxorubicin, Vincristine, Prednisone; R-GemVin, Rituximab, Gemcitabine, Vinorelbine; R-ICE, Rituximab, Ifosfamide, Carboplatin, Etoposide; SCT, Stem cell transplantation; R-EPOCH, Rituximab, Etoposide, Prednisone, Vincristine, Cyclophosphamide, Doxorubicin. † Patient deceased. → Response ongoing at last follow-up.

The case presented by Pang et al. Patient achieved CR with ibrutinib but experienced relapses each time treatment was interrupted, even after only a few days of discontinuation (33). Consequently, sustained CR was only achieved with continuous ibrutinib therapy, displaying the sensitivity of PCDLBCL-LT to BTKi, even when lower doses of this medication are used (140 mg daily) (33). Overall, ibrutinib has shown promising results in treating R/R PCDLBCL-LT, with various combinations and dosing strategies yielding positive outcomes in several reported cases. In our patient, MYD88 mutation analysis could not be performed.

First-generation BTKi, particularly ibrutinib, are associated with significant off-target toxicities, including an increased risk of bleeding, atrial fibrillation, and hypertension. In our case, the patient was already receiving a DOAC, compounding the bleeding risk and necessitating a more selective therapeutic approach, additionally patient had a high cardiovascular risk due to age and history of hypertension prompting for a different BTKi alternative. Other considerations included the possible association with CLL and Richter’s transformation which supported the use of a BTKi. Second-generation BTKi, such as acalabrutinib and zanubrutinib, have been developed to offer greater target specificity and improved safety profiles (12, 13). However, to date, there have been no published reports documenting the use of either agent (Acalabrutinib or Zanabrutinib) in PCDLBCL-LT.

Our report describes, to our knowledge, the first documented case of successful treatment of relapsed/refractory PCDLBCL-LT with acalabrutinib. The patient achieved a complete and durable response without significant toxicity, highlighting the potential role for second-generation BTKi in this rare and aggressive cutaneous lymphoma. This case contributes to a growing body of literature supporting further evaluation of next-generation targeted agents in relapsed/refractory PCDLBCL-LT.

## 4 Conclusion

We present the remarkable clinical activity of acalabrutinib in a case of R/R PCDLBCL-LT with rapid clinical progression and no adverse events. PCDLBCL-LT is highly refractory, and novel therapy strategies are needed, mainly for patients with R/R disease.

Large multicenter clinical trials may be too difficult to conduct. However, basket trails or collaborative multicenter studies would be more feasible and are needed to define standards of care in PCDLBCL-LT, particularly to evaluate BTKi combinations and the early introduction of these agents in frontline management. A multidisciplinary management approach and precise diagnosis, including molecular testing, will benefit patients with PCDLBCL-LT and facilitate the tailored selection of treatment strategies to improve patient outcomes.

## Data Availability

The raw data supporting the conclusions of this article will be made available by the authors, without undue reservation.
